# Digital technology for the prevention of healthcare-related infections in critical care

**DOI:** 10.1590/0034-7167-2022-0528

**Published:** 2023-11-13

**Authors:** Letícia Pontes, Bárbara Alessandra Tibério, Jéssica de Fátima Gomes Pereira, Renata Rodrigues da Luz

**Affiliations:** I Universidade Federal do Paraná. Curitiba, Paraná, Brazil.

**Keywords:** Technology, Infections, Health Education, Critical Care, Family, Tecnología, Infecciones, Educación en Salud, Cuidados Críticos, Familia, Tecnologia, Infecções, Educação em Saúde, Cuidados Críticos, Família

## Abstract

**Objective:**

To develop digital technology for patient and family integration into the Intensive Care Unit care team, aiming to subsidize decision-making for the prevention of infections related to healthcare.

**Method:**

Methodological research of technological production in three phases: pre-production, production, and post-production in a teaching hospital in southern Brazil. Sixteen intensive care unit nurses participated.

**Results:**

The research produced six videos: general guidelines on the Intensive Care Unit, Preventing infections: hand hygiene; Pneumonia associated with mechanical ventilation; Catheter-associated primary bloodstream infection; Catheter-related urinary tract infection.

**Final considerations:**

The proposed technology was developed and aims to assist nurses in bringing patients and families closer to the routines of the intensive care environment to provide safety in the contact of the patient of intensive care units with family members and in the active participation for the prevention of infections related to healthcare.

## RESUMO

**Objetivo:**

Desenvolver tecnologia digital para integração de paciente e família à equipe de cuidados da Unidade de Terapia Intensiva, visando subsidiar a tomada de decisão para prevenção das infecções relacionadas à assistência à saúde.

**Método:**

Pesquisa metodológica, de produção tecnológica, em três fases: pré-produção; produção; pós-produção, em hospital de ensino no Sul do Brasil. Participaram 16 enfermeiros de Unidade de Terapia Intensiva.

**Resultados:**

Produziram-se seis vídeos: Orientações gerais sobre Unidade de Terapia Intensiva; Prevenindo infecções: higienização das mãos; Pneumonia associada à ventilação mecânica; Infecção primária da corrente sanguínea associada ao cateter; Infecção do trato urinário relacionada ao cateter.

**Considerações finais:**

A tecnologia proposta foi desenvolvida e visa auxiliar enfermeiros na aproximação de pacientes e familiares às rotinas do ambiente de cuidados intensivos, para proporcionar segurança no contato do paciente de Unidades de Terapia Intensiva com familiares e na participação ativa para prevenção de infecções relacionadas à assistência à saúde.

## RESUMEN

**Objetivo:**

Desarrollar tecnología digital para integración de paciente y familia al equipo de cuidados de Unidad de Cuidados Intensivos, objetivando subsidiar a la toma de decisión para prevención de infecciones relacionadas a la atención de salud.

**Método:**

Investigación metodológica, de producción tecnológica, en tres etapas: preproducción; producción; postproducción, en hospital de enseñanza en el Sur Brasileño. Participaron 16 enfermeros de Unidad de Cuidados Intensivos.

**Resultados:**

Producidos seis vídeos: Orientaciones generales sobre Unidad de Cuidados Intensivos; Previniendo infecciones: higiene de manos; Neumonitis relacionada a ventilación mecánica; Infección primaria de la corriente sanguínea y Infección del tracto urinario relacionadas al catéter.

**Consideraciones finales:**

La tecnología propuesta desarrollada objetiva auxiliar enfermeros en el acercamiento de pacientes y familiares a rutinas del ambiente de cuidados intensivos, para proporcionar seguridad en el contacto del paciente de Unidades de Cuidados Intensivos con familiares y participación activa para prevención de infecciones relacionadas a la atención de salud.

## INTRODUCTION

The Intensive Care Unit (ICU) is a complex unit differentiated from other inpatient units because it is an environment predominantly equipped with technological resources and has work dynamics that require specific skills and knowledge. The ICU offers treatments that are considered aggressive/invasive and can generate emotional and psychological disorders both for the patient and for his family and the professionals involved. Therefore, critical patient care should be individualized, humanized, and welcoming. In addition, patient-and family-centered care provides better results in treatment and recovery^(^ 1 ^)^.

Thus, a new perspective to develop and consolidate the principle of humanization in health services has received strength and a new look has turned to care, through the provision of quality services, based on effective practices to ensure patient safety. In 2000, the American report “ To err is human: building a safer health care system” pointed out that millions of people suffer injuries and deaths as a result of health practices worldwide. The publication of this report started initiatives to promote patient safety, understood as a strategy that seeks to reduce, to the minimum acceptable, the risk of unnecessary harm related to healthcare^(^ 2 ^)^.

Among these damages, healthcare-related infections (HCRI) stand out, which are characterized as the most frequent adverse events in this care. Its occurrence increases morbidity, mortality, and hospital costs, in addition to affecting patient safety and the quality of health services^(^ 3 ^)^.

The National Patient Safety Program (NPSP), whose objective is the qualification of health care, highlights the involvement of patients and families in care actions, meeting the MS/GM Ordinance No. 529/2013, which considers the involvement of patients and families as a strategy for their safety^(^ 4 ^)^.

When considering the family and the patient as subjects of care who must be incorporated into the multidisciplinary ICU care team, the research question emerged: “how to integrate the patient and family into the ICU care team and include them in decision-making for the prevention of infections related to healthcare”?

## OBJECTIVE

To develop digital technology for patient and family integration into the Intensive Care Unit care team, aiming to subsidize decision-making for the prevention of HCRI.

## METHODS

This is methodological research of technological production, developed in a graduate program, in the modality of a professional master, in the Technology and Innovation in Health (TIS) research group, from August 2019 to June 2020. The research was conducted in a teaching hospital in the Southern Region of Brazil, specifically in the adult intensive care unit. It offers 14 beds to receive patients over the age of 14. It assists, on average, 42 patients/month by clinical and surgical criteria of various specialties such as medical clinic, neurology, pulmonology, and infectious diseases.

The research followed the ethical aspects provided for by Resolution No. 466/2012 of the National Health Council (CNS) and approved by the Research Ethics Committee of the Hospital de Clínicas complex of UFPR by Opinion No. 3,615,856.

**Figure 1:**
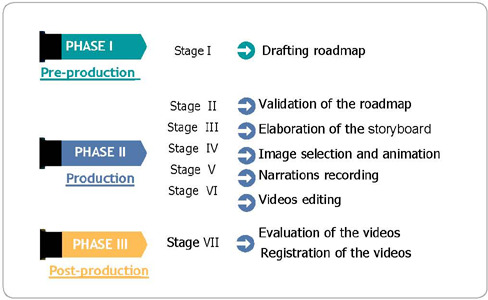
Phases and methodological stages for the creation of the technology

For the creation of the technology, the research followed three phases and seven stages (Figure 1).

Initially, the research theme was defined as the integration of the individual under care of a multidisciplinary team in the ICU and his family into decision-making for the prevention of infections related to healthcare. Then, it established that the objective of the videos was to function as a tool to support the multidisciplinary team, in the orientation of patients and families, about the prevention process of infections related to healthcare. The videos content was also defined at this stage.

### Phase I: Pre-production

#### Stage 1: Drafting the roadmap

To prepare the first version of the video roadmap, the information contained in the program guidelines “Improving Patient Safety on a large scale in Brazil” was adopted as a starting point^(^ 5 ^)^. Still, the researchers conducted a literature review in the database to achieve the proposed objective: Latin American & Caribbean Health Sciences Literature (LILACS), Medical Literature Analysis and Retrieval System Online (MEDLINE), and nursing database (BDENF). For the search strategy, the analysis used descriptors in health sciences (DeCS): Educational Technology OR *Tecnologia Educacional* AND Nursing Care OR *Cuidados de Enfermagem* AND Family OR *Família* AND Intensive Care Units OR *Unidades de Terapia Intensiva*. It included primary studies published between 2013 and 2019, which addressed the theme “integration between patient, family, and ICU care team, for the prevention of care-related infections.” The time frame occurred due to the period of the creation of Ordinance MS/GM nº 529/2013, which considers the involvement of patients and families as a strategy for patient safety. After analyzing the findings, the themes were separated by topics; and the information was summarized in an illustrated roadmap to facilitate understanding and streamline reading.

### Phase II: Production

This phase included the stages of validation of the content of the previously prepared roadmap, preparation of storyboards, image selection and animation production, narration recording and video editing.

#### Stage 2: Validation of the roadmap by participants

This step aimed to ensure the quality of the information and detect weaknesses in the roadmap of the videos. The inclusion criterion of the collaborators who would validate the roadmap content was: being a nurse based at the ICU. The exclusion criterion was: being away from professional activities during the data collection period. One of the researchers invited the nurses individually to participate in the study. The researcher presented the theme, the objectives and procedures to which they would be submitted. Those who agreed to participate in the study were asked to sign an Informed Consent Form.

The sample of participants was 16 nurses. The validation roadmap was presented to the collaborators, along with an instrument prepared by the researchers, with the help of the Google Forms tool, and included identification data, academic education, and professional experience, followed by seven propositions: In your perception, the integration of the family into the ICU care team is important; As for the content roadmap presented, the topics addressed are pertinent the ICU routines; As for the content presented regarding the routines in the ICU, you; As for the content presented regarding hand hygiene, you; As for the content presented regarding care with the central venous catheter, you; As for the content presented regarding care with indwelling urinary catheter, you; As for the content presented regarding care with the orotracheal tube, you. The answers to these propositions were: agree; partially agree; neither agree nor disagree; partially disagree; and disagree.

The research used the Content Validity Index (CVI) for each proposition. The propositions that obtained a minimum concordance of 0.80 were considered validated. Items that did not reach the minimum index were reviewed, and all recommendations were fully met and maintained in the roadmap. Additional suggestions could also be listed in a specific field at the end of each preposition to make the information clearer, more objective, and aligned with the reality of the research field.

The participants indicated some recommendations to address in the videos, such as the importance of hand hygiene always when performing care; maintenance of the patient’s identification bracelet; orotracheal aspiration procedure and tube fixation; use of masks and aprons, when necessary; non-manipulation of the devices and dressings by the family member; catheter protection during bathing; among others. Researchers followed these recommendations for the elaboration of the storyboards.

#### Stage 3: Elaboration of storyboards

After making the necessary adjustments in the roadmap, based on the contributions of the collaborators in the validation, the elaboration of the storyboards, which consists of creating a frame with two columns: the first, with the description in chronological order of the images (figures, photos, schemes) contained in the video; and the second column, with the text to be narrated, accompanied by a background music.

#### Stage 4: Image selection and animation production

At this stage, research selected the following visual elements: multidisciplinary team; intensive care environment; orotracheal intubation procedures; medical devices; among others. The objective of the images was to present, in the videos, the information clearly and attractively. The image search took place in the image bank Freepik (paid license premium), Pngtree and Pixabay (free license), and Google Images, respecting copyright rules.

#### Stage 5: Narrations recording

With the storyboards produced in the previous stage, the narrations were recorded in an acoustically isolated studio and then edited by a professional in the sounding area.

#### Stage 6: Editing

The Animaker application helped in the edition of the videos, which offers animation features, scenarios and allows upload of still images and audio files. After the scenes were finished, the first version of the videos was exported to a digital file.

### Phase III: Post-production

The third phase, called post-production, involved the final stage of evaluation of the first version of the videos by a group of judges and registration with the National Film Agency (ANCINE).

#### Stage 7: Evaluation of the videos

The videos were submitted for evaluation by a group of judges who needed to meet the following criteria: be a full-time nursing professional in the adult ICU and work in the care of critical patients. The evaluation instrument included ten propositions^(^ 6 ^)^ about the videos, for which the available answers were: agree, partially agree, neither agree nor disagree, partially disagree, and disagree. Additional suggestions could be listed in a specific field at the end of the instrument. The propositions were: a) “the videos have language that is easy to understand;” b) “the audio resource is properly used;” c) “the content was distributed appropriately for the duration of the video;” d) “it facilitates the memorization of messages;” e) “the visual resources are properly used: the images chosen are easy to understand;” f) “it transmits the proposed guidelines;” g) “maintains audience throughout the duration;” h) “the information about the contents addressed is correct;” i) “a person with poor reading ability understands information;” J) “it may facilitate guidance in integrating the family into the ICU;” and there was space for additional suggestions. The evaluation instrument was prepared in Google Forms and sent to the judges by email, along with the video links.

## RESULTS

### Profile of participants

Sixteen professional nurses from the ICU participated in this research. Of these, 75% were female, aged between 30 and 47. Concerning the length of professional training, the period from eleven to twenty years predominated, and the time of ICU work from one to ten years. The group of professionals participated in the validation of the content of the roadmap and the evaluation of the videos.

### The roadmap for the production of the videos

The roadmap called “Understanding the ICU” provide tion about the purpose, characteristics of the ICU and care to which patients in intensive care are submitted; on aspects related to patient safety, highlighting inf related to the care needed to prevent HCRI.

### Validation of the roadmap content

The analysis evaluated six items. For each of them, it the CVI according to Table 1.

**Table 1:** Validation of the roadmap, Curitiba, Paraná, Brazil, 2022

Nº	Evaluated Items	CVI %
01	As for the content roadmap presented, are the topics addressed pertinent to the ICU routine?	0.9
02	As for the content presented regarding routines in the ICU, you…	0.8
03	As for the content presented regarding hand hygiene, you…	0.9
04	As for the content presented regarding care with the central venous catheter, you…	0.7
05	Regarding the contents presented regarding the care with indwelling urinary catheter, you…	0.8
06	Regarding the content presented regarding care with the orotracheal tube, you…	0.9

*IVC: Content Validity Index.*

Of the six items presented, five had CVI betwee 0.8. The item “As for the content presented regarding central venous catheter” reached a CVI of 0.7 and dese sion. Therefore, the content was adequate, and the ob of the expiration date of the dressings was excluded from the roadmap since the dressing change may vary according to the type of coverage applied on the catheter, which may generate an interpretation error by the family member.

### Editing the videos

Six videos were produced in 2D animation, with an average duration of two minutes and 12 seconds. The option for conversational language followed the recommendations of multimedia learning. The scenes were elaborated in the Animaker application, according to the principles of multimedia education to promote better understanding of the content by patients and the family.

**Figure 2:**
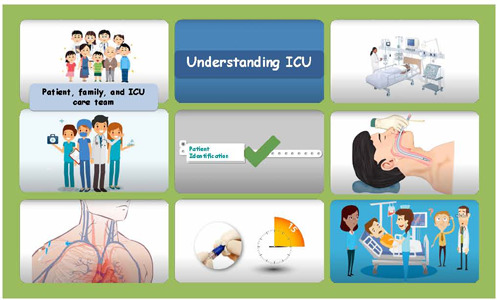
Scenes from the videos

The videos were named: General guidelines on the Intensive Care Unit; Preventing infections: hand hygiene; Pneumonia associated with mechanical ventilation; Catheter-associated primary bloodstream infection; Catheter-associated urinary tract infection. Figure 2 shows some scenes of the videos produced.

### Evaluation of the videos

A group of eleven nurses, who also participated in the validation of the roadmap content, evaluated the videos. The non-participation of five nurses in this research stage is justified by their relocation to other sectors momentarily due to the pandemic conditions faced at the study site. The judges evaluated the six videos.

For evaluation, they were asked to respond to the ten statements on a five-point scale, in which 1, the professional agreed with the statement; and 5, disagreed. The statements evaluated are shown in Table 2.

**Table 2:** Judges’ Evaluation

Evaluated statements	I agree %	I partially agree %	Indifferent %	I Partially disagree %	Disagree %
The videos feature language that is easy to understand.	100	-	-	-	-
The audio feature is properly utilized.	90.9	9.1	-	-	-
The content was properly distributed for the length of the video.	90.9	9.1	-	-	-
It facilitates the memorization of messages.	90.9	-	9.1	-	-
The visual aids are effectively used: the selected images are easy to understand.	81.8	9.1	9.1	-	-
It transmits the proposed guidelines.	90.9	9.1	-	-	-
It keeps the audience throughout the duration.	100	-	-	-	-
The information about the contents covered is correct.	80	20	-	-	-
A person with poor reading ability understands information.	81.8	18.1	-	-	-
It may facilitate guidance in integrating the patient and family into the ICU.	100	-	-	-	-

*(-) Numeric data equal to zero, not resulting from rounding.*

The judges’ commitment to the research was not only to evaluate the videos but, also, to suggest how they could be used in the unit. The judges recommended making the videos available in the waiting room in the ICU while family members wait to visit. The evaluation by the target audience (patient and family) was not possible due to the covid-19 pandemic.

Finally, the videos were registered as a serial work of 2D animation videos, called “Patient, Family, and ICU Care Team: Together in the Prevention of Infections” with a Brazilian product certificate of Nº B20-004931-00000 – ANCINE.

## DISCUSSION

The proposal to develop digital technology to integrate patients and families into the ICU care team resulted in the production of a series of videos that covers everything from general guidelines on the ICU to the main procedures and measures for the prevention of infections related to healthcare.

Because they are developed to contribute to the patient and family integration into the ICU care team, the research meets the idea that the family can help the patient adhere to treatment and in the care process. Therefore, qualified reception is necessary, with an approach to issues relevant to the hospitalization in a critical environment^(^ 1 ^)^.

The importance of family participation in critical care units is observed in both adult and pediatric intensive care settings. A study conducted in Belo Horizonte, in the neonatal ICUs of a public maternity hospital, found that the insertion of family members as critical and active partners in the practices of health professionals is a promising strategy for the promotion of patient health and safety^(^ 2 ^)^.

The videos produced, which evaluators considered appropriate, offer clear and easy-to-understand language. Videos are deemed significant technologies as support in health education processes because, in addition to formal content, they contain images and audio that facilitate access to information, allowing the public to reach the message, which often the health professional cannot transmit^(^ 7 ^)^.

To use technology for health education, research conducted in Brazil aimed to develop a website on the predominant infections related to healthcare and the respective bundles for the prevention of such infections in intensive care units. The authors believe that the connection of technologies with educational practices has subsidized the process of disseminating information related to the prevention of HCRI^(^ 8 ^)^.

The information presented in the videos shows aspects related to the general guidelines on ICU, which address hand washing, infections associated with mechanical ventilation, and the use of catheters. The content describes to the patient and his family the reality of care in the ICU. In Malaysia, for a similar purpose, researchers created and validated an information page for relatives of ICU patients. This page can help family members with information related to the ICU environment and facilities, support services, staff roles, medication and procedures, nursing care, and ICU transfer^(^ 9 ^)^.

The creation of digital technologies can be a valuable and viable resource for family members to support and reinforce the learning process, helping them to understand the intensive care environment^(^ 10 ^)^. This statement is in line with the purpose of the videos produced, to serve as an accessible tool capable of facilitating the transmission of guidelines relevant to the care developed in the ICU.

### Study limitations

Despite the positive evaluation of the videos by ICU nurses, the impossibility of evaluation by the target audience due to the covid-19 pandemic, is considered a limitation of the study.

### Contributions to the fields of Nursing, Health, or Public Policy

The videos will promote the engagement of the family with the ICU care team, according to the evaluation of the judges. They can contribute to the knowledge and appreciation of measures for infection control and patient safety practiced in the intensive care environment.

## FINAL CONSIDERATIONS

According to the evaluators of the technology developed, the videos have the adequate duration and offer clear language and narration, easy to understand. Visual aids are properly used, with images that convey the proposed message. They also conclude that the guidelines can contribute to integrating the family into the ICU care team.

The development of this technology is characterized as an innovation in the health education process, with the possibility of assisting nurses in approaching patients and their families to the routines of the intensive care environment. It has the potential to reach a large audience and meet the needs of this, especially of people with reading difficulties, because the videos are stored on YouTube channel, accessible to those with visual impairment since the content is narrated, and with hearing impairment, since there is the option of viewing subtitles.

Access to the videos is possible via the link: https://www.you-tube.com/playlist?list=PLtXx9QMCpn0dQR4YZVIyd5tfVrdRcxmlR

The videos do not replace the nurses’ role in the process of integrating patients and families into the care team. The content of the digital technology developed in this work addresses general guidelines, but each patient presents specific needs and realities. Therefore, it is up to the nurse to detect them and adjust the guidelines to ensure the adherence of patients and families and, consequently, make them partners in the care process.

The study recommends the implantation of the technology to assess the impact in practice through intervention studies.
